# “Bend so you don’t break!” A longitudinal moderated mediation study on human resources management practices, humility, psychological well-being, and job performance

**DOI:** 10.3389/fpsyg.2024.1452848

**Published:** 2024-10-17

**Authors:** Annick Parent-Lamarche, Julie Dextras-Gauthier

**Affiliations:** ^1^Department of Human Resource Management, Université du Québec à Trois-Rivières, Trois-Rivières, QC, Canada; ^2^Department of Management, Laval University, Quebec, QC, Canada

**Keywords:** human resources management practices, humility, psychological well-being, job performance, longitudinal, moderation, mediation, moderated mediation

## Abstract

**Introduction:**

This study aims to examine the mediating role of psychological well-being in the relationships between human resources management practices and job performance. Also, this study aims to assess the moderating role of humility on these relationships.

**Methods:**

Multiple regression, mediation, and moderation analyses were conducted with MPlus software on a sample of 569 workers who filled out a questionnaire at both Time 1 and Time 2. Both data collections took place between April 20, 2022, and May 2, 2022, for Time 1, and between June 20, 2022, and July 3, 2022, for Time 2. Data were collected through the Leger Opinion (LEO) online panel, with respondents required to be workers.

**Results:**

We found that psychological well-being at T1 did not play a mediating role between human resources management practices at T1 and job performance at T2. Also, humility did not moderate the relationships between human resources management practices at T1 and psychological well-being at T1 but did significantly moderate the longitudinal relationships between human resources management practices at T1 (i.e., dotation, formation, career management, autonomy, occupational health and safety, diversity management, indirect compensation, flexibility, performance management), and job performance at T2.

**Discussion:**

For all significant interactions, the results indicated that when humility was high, the longitudinal effect of good human resources management practices led to high in-role job performance.

## 1 Introduction

Human resource management (HRM) is defined as a set of practices for planning, directing, organizing, recognizing, and developing human resources within an organization (St-Onge et al., 2021). HRM encompasses various practices associated with HR activities, including recruitment, training, compensation management, performance management, and more (St-Onge et al., 2021). Initially, the goal was to ensure organizational performance according to the High-Performance Work System (HPWS) (Kaushik and Mukherjee, 2022). However, it is now essential to ensure that these practices not only avoid harming psychological well-being but also have the potential to enhance it (Guest, 2017).

Regarding psychological well-being, it is a broad concept that encompasses two primary dimensions: hedonic and eudaimonic. One of the most widely recognized definitions of well-being was proposed by Ryan and Deci (2001), emphasizing optimal experiences and functioning. The hedonic dimension focuses on happiness, viewing well-being as the pursuit of pleasure and the avoidance of pain (Ryan and Deci, 2001). In contrast, the eudaimonic dimension relates to finding meaning in life and achieving self-realization (Ryan and Deci, 2001). For its part, the World Health Organization (WHO) defines and measures well-being in relation to factors such as mood, vitality, and personal interests (Topp et al., 2015). Research has shown that low levels of employee psychological well-being are negatively associated with job performance (Sonnentag, 2015). Given the significance of employee well-being, this variable has garnered considerable attention from researchers studying various organizations and sectors (Steel et al., 2018). That said, the impact of HRM practices on psychological well-being (e.g., Alfes et al., 2012), as well as the mediating effect of psychological well-being on job performance (Salas-Vallina et al., 2021), has received less attention compared to the research focused on the effect of HRM practices on job performance (e.g., Snape and Redman, 2010).

Indeed, job performance has been the focus of extensive research and continues to be a prominent topic in organizational studies (Carpini et al., 2017). Job performance notably includes in-role behaviors (Katz, 1964; Williams and Anderson, 1991). In-role job performance refers to the specific duties and responsibilities associated with completing an employee’s tasks (Rotundo and Sackett, 2002). Essentially, in-role job performance involves meeting established performance standards (Katz, 1978) and is formally recognized as a core component of the job (Rotundo and Sackett, 2002).

To this day, the conceptualization and measurement of HRM practices is still lacking consensus and clarity (Peccei and Van De Voorde, 2019). Various relationships between HRM practices, well-being, and performance conceptualizations are described in the literature (Peccei and Van De Voorde, 2019). These relationships can be distinguished based on the type of mediation effect that well-being has (Peccei and Van De Voorde, 2019). Human resources management is expected to have a favorable effect on job performance via employee well-being according to a mutual-gains perspective (win–win scenario) (Peccei and Van De Voorde, 2019). Conversely, HRM is believed to have an unfavorable effect on employee well-being when job performance is attained at the sacrifice of employee well-being according to the conflicting-outcomes perspective (win–lose scenario) (Peccei and Van De Voorde, 2019). Alternatively, HRM is likely to be negatively associated with employee well-being, and this unfavorable relationship is expected to reduce job performance according to the mutual-losses perspective (lose–lose scenario) (Peccei and Van De Voorde, 2019). As Peccei and Van De Voorde (2019) mention, the relationship between HRM and well-being is poorly understood, and more systematic attention must be paid to it to strengthen the theoretical underpinnings of HRM, well-being, and job performance.

In the current context of labor shortage (Ferguson and Makinizi, 2023), employers are interested in focusing on HRM practices that promote a positive image of their organizations. One way to do so is to ensure that their actions will be in alignment with a mutual-gains perspective (win–win scenario). Furthermore, from an ethical point of view, organizational performance should not be achieved at the expense of individual well-being; on the contrary, it should rely on it (Guest, 2017). However, the dominant theoretical models and empirical research in HRM continue to emphasize ways to improve performance through HRM practices from an organizational perspective (Guest, 2017). Thus, the perspective and well-being of employees are seen as secondary concerns (Boxall et al., 2016). A new HRM practices scale known as the High Wellbeing and Performance Work System (HWBPWS), which is based on the integrated mutual-gains model of Guest (2017), was developed and validated (Parent-Lamarche et al., 2023). That said, the predictive capacity of this new scale has never been verified. Given that this scale was developed based on Guest’s model (Guest, 2017) to enhance well-being as a precursor to performance, it is expected that all practices will have a positive effect on employees. Consequently, this study’s first objective is to examine whether the ten HRM practices included in the HWBPWS are conducive to employee psychological well-being, which later translates into job performance (win–win scenario). In other words, this study’s first objective is to examine the mediating role of psychological well-being in the relationship between HRM and job performance.

Alongside HRM practices (i.e., job resources), there are also individual resources that could differentiate patterns of behavior or attitudes among the human resources within organizations (Grover et al., 2018). It seems important to identify the cumulative and interactive impact of different levels of resources (Nielsen et al., 2018). This could pave the way to a more comprehensive reflection on the implementation of HRM practices aimed at creating healthy working environments. Employers could deploy practices that would allow their employees to grow individually and develop their own resources and strengths. Because individuals perceive and cope with their environments differently (Lazarus and Folkman, 1984), psychological well-being and performance may differ among employees facing the same HRM practices. In addition, studies focusing on the development of individual resources remain necessary to ensure the adaptation of employees to the new realities that await them (Potgieter et al., 2019). One resource that appears to be important in competitive and, therefore, potentially ego-threatening work environments is humility, which is considered a virtue and a human strength (Peterson and Seligman, 2004). In positive psychology, humility is seen as a predictor of human excellence and flourishing (Peterson and Seligman, 2004).

According to Owens et al. (2013), humility (i.e., expressed humility) is an individual characteristic that emerges in social interactions, and it has three dimensions: 1. accurate self-awareness, 2. an appreciation of others’ strengths and contributions, and 3. teachability. The first dimension entails an aspiration to engage in a process of accomplishing authentic self-awareness via interactions with others. In this regard, individuals who can preserve realistic self-views tend to be more psychologically healthy and have higher general well-being (Vaillant, 1992). Humble individuals attempt to gain authentic or accurate reflection of themselves via others by being transparent about their strengths and limitations (Owens et al., 2013). The second dimension captures attitudes that are other enhancing instead of self-enhancing (Morris et al., 2005). Indeed, humble individuals have balanced perceptions that recognize both strengths and limitations and do not attempt to under-or overrepresent themselves (Morris et al., 2005). The third dimension reflects the tendency to approach interpersonal interactions with an objective of learning via others, which is manifested by showing openness to retroaction, advice, and alternative ideas (Owens et al., 2013). Humility is a strength that makes it possible to overstep the comparative–competitive response when interacting with others and, instead, accept, recognize, and appreciate their qualities and contributions without feeling threatened (ego threat) by them (Exline et al., 2004).

Humility should be distinguished from modesty and both narcissism types [i.e., 1. the grandiose type, which is characterized by inflated self-image, entitled attitudes, feelings of superiority, interpersonal manipulation, domineering behavior, fantasies of unlimited power, a need for admiration, self-assuredness, extraversion, and social competence (Pincus and Roche, 2011)], and 2. the vulnerable type, which can be characterized by entitled attitudes, a need for admiration, helplessness, shame, emptiness, low self-esteem, hypersensitiveness, defensiveness, proneness to anxiety, and depression (Pincus and Roche, 2011). Indeed, humility can temper inflated egos and arrogance, which are often associated with narcissism–either the grandiose or vulnerable type, and consequently facilitate engagement and learning (Li, 2016; Tangney, 2000), as well as ensuring a healthy ego/healthy narcissism, which is characterized by assertiveness, a positive self-image, appropriate ambition, empathy, and commitment (Pincus and Lukowitsky, 2010). Humility is also theoretically associated with a stable or tempered self-view that does not over-inflate (e.g., arrogance and superiority) with praise or over-deflate (e.g., shame and self-hate) with criticism (Owens et al., 2013), which is the case with narcissistic individuals. Also, humility does not equate to a lack of self-esteem, weakness, submissiveness, or unassumingness (Nielsen and Marrone, 2018; Owens et al., 2013). On the contrary, high self-esteem is required to express humility without experiencing significant ego threat. Humility can sometimes be confused with shame or self-diminishment, but it indicates a strong ego, which allows individuals to cope with the limitations of the self (Sandage et al., 2017). Modesty, which refers to underselling accomplishments, lacking assertiveness, or withholding positive information about the self, does not imply the motivation to engage in personal learning and development, which is an important component of humility (Owens et al., 2013). Additionally, modesty is sometimes seen as a response to situational demands or pressures and, therefore, considered an impression-management tactic (Peterson and Seligman, 2004). Narcissism, in general, is defined as a sense of grandiosity, arrogance (despite insecurity), self-absorption, entitlement, fragile self-esteem (sometimes self-hate), the constant interplay of excessive pride and shame, deceitfulness, envy, rage commonly known as narcissistic rage (Delisle, 1993; Krizan and Johar, 2015), and hostility (Czarna et al., 2018; Pincus and Roche, 2011). However, a lack of those characteristics does not equate the presence of humility (Tangney, 2000; Zhang et al., 2017). That said, narcissism is an antecedent of a lack of humility (Sandage et al., 2017; Tangney, 2000), meaning that narcissistic individuals, especially the vulnerable type, are the most likely to lack humility, even though they can falsely display modesty as a self-presentation stratagem (Brown and Brunell, 2017). In contrast, attachment styles, forgiveness [i.e., the capacity to react to interpersonal harm and, sometimes, misperceived harm by regulating urges for vengeance and avoidance, such as the silent treatment, and, instead, responding in a prosocial manner that involves communication (Sandage et al., 2017)], and resilience are documented as antecedents of humility (Nielsen and Marrone, 2018). In other words, simply not being a narcissist does not mean that one is humble, but being a narcissist typically means that one is not humble.

Therefore, a person must have the humility to “bend” to avoid an ego “break” and the anticipated consequences on psychological well-being and job performance. A fragile ego will prevent one from being humble enough to “bend,” and that is what will ultimately “break” an individual. This must be especially true in the increasingly competitive and performance-driven world of work. Because of this, humility appears to be important at the organizational level but also at the individual level. Indeed, it promotes personal development and the achievement of objectives, in addition to protecting one’s ego and those of others a healthy ego/humility is less likely to induce suffering in others and cause a vicious cycle of narcissistic wounds, leading to less humble individuals in time (Behary, 2021). This may also help maintain favorable interpersonal relationships and a good working climate for the benefit of all, including employers. Indeed, humble people with a healthy ego/healthy narcissism do not tend to disappear within an abyss of silence, which is more typical of others who lack humility, have an unhealthy ego, or engage in unhealthy narcissism (Behary, 2021). Consequently, our second objective is to study the moderating role of humility as an individual resource that is likely to influence the capacity for adaptation at work. Indeed, this study’s second objective is to examine the moderating role of humility on the relationships between HRM practices and psychological well-being, as well as between HRM practices and job performance.

Four main gaps in the existing literature justify these two objectives: 1. the lack of studies on employee humility because the studies almost all focus exclusively on leaders, 2. the absence of studies examining the interactions between HRM practices and employee humility, 3. the lack of studies on their (i.e., interactions between HRM practices and employee humility) subsequent effects on psychological well-being and job performance over time, and 4. the absence of an empirical study aimed at examining the predictive capacity of the new scale known as the High Wellbeing and Performance Work System. Human resources management was expected to have a favorable effect on job performance via employee psychological well-being according to a mutual-gains perspective (win–win scenario). It is crucial to thoroughly understand the impact of HRM practices on these outcomes, as well as the moderating role of humility, to better address employees’ needs in the future. This understanding may involve improving HRM practices that do not meet expectations regarding these outcomes or capitalizing on those that do. Furthermore, we should not overlook the importance of supporting individual resources in employees, particularly through training programs.

## 2 Hypothesis development

The theoretical model proposed in this study is primarily based on the integration of various frameworks, including human resource models, organizational psychology models, and personality psychology models. Specifically, it incorporates: The Integrated Mutual-Gains Model (Guest, 2017) from human resource management, Peterson and Seligman’s Character Strengths and Virtues (CSV) framework (Peterson and Seligman, 2004; Peterson and Seligman, 2004) and the HEXACO (Ashton and Lee, 2020) model from personality psychology, and The Conservation of Resources Model (Hobfoll, 1989), and the Job Demands-Resources Model (Demerouti et al., 2001) from organizational psychology. These models complement one another and deepen our understanding of psychological well-being and performance in the workplace, additionally concerning the role of employee humility in these relationships.

The integrated mutual-gains model is based on the premises of social exchange theory (Blau, 1968; Cropanzano and Mitchell, 2005), according to which high psychological well-being leads to high job performance. Similarly, employees who enjoy a high level of psychological well-being perform well, and vice versa, according to the “happy worker–productive worker” thesis (Warr and Nielsen, 2018). Additionally the conservation-of-resources model (Hobfoll, 1989) supposes that benefiting from a great deal of resources increases the capacity to face future stressful situations and is also a predictor of employee psychological well-being, which increase the motivation to perform (Ryan and Deci, 2000). However, these frameworks do not indicate specific HRM practices that will lead to high levels of psychological well-being and, consequently, performance. As stated by Nielsen et al. (2017), the “happy worker–productive worker” thesis does not indicate the antecedents of such states, which limits the potential for actions on the part of organizations. Guest (2017) offers more guidance and proposes that there are five essentials upstream of psychological well-being: (1) investing in employees, (2) providing engaging work (i.e., stimulating work), (3) a positive social and physical work environment, (4) voice (i.e., encouraging employee participation), and (5) organizational support. The HRM practices comprised in these five essentials are supposed to lead to high job performance via the psychological well-being of employees. Accordingly, these practices are therefore expected to be equally beneficial. This approach is coherent with a mutual-gains perspective (win–win scenario).

To our knowledge, no study has attempted to empirically verify Guest’s model. However, a few empirical studies have considered the effects of HRM practices on various outcomes, including job performance. One unpacked the social mechanisms involved via well-being-oriented HRM practices, which increased resilience and subsequent employees’ performance (Cooper et al., 2019). Another study found that positively perceived HRM practices were associated with increased citizenship behaviors and well-being and lower turnover intentions (Alfes et al., 2012). Regarding the mediating role of psychological well-being, it was previously found that employee well-being partially mediated the relationship between the perceived use of skill-and opportunity-enhancing HR practices and in-role job performance (Khoreva and Wechtler, 2018). That same study also found that employee well-being partially mediated the relationship between the perceived use of motivation-enhancing HR practices and innovative job performance (Khoreva and Wechtler, 2018). However, to our knowledge, no study specifically taps into the potential mediating role psychological well-being could play in the relationships between the HRM practices that derive from Guest (2017) model and job performance. As mentioned in the introduction, HRM practices aligned with Guest’s model (2017) should enhance well-being as a precursor to performance; therefore, it is expected that these practices will positively impact employees’ psychological well-being, ultimately leading to higher job performance over time. Consequently and in accordance with the empirical background, we propose the following hypothesis:

*H1*: Psychological well-being at T1 plays a mediating role in the relationships between HRM practices at T1 and job performance at T2.

According to various models and empirical literature, beyond HRM practices, individual characteristics are also likely to play a role in the relationships between HRM practices, psychological well-being, and performance.

Peterson and Seligman’s Character Strengths and Virtues (CSV) framework is a classification system developed to understand positive traits that contribute to human flourishing (Peterson and Seligman, 2004). It serves as a counterpart to traditional models of psychological disorders, focusing on strengths rather than weaknesses (Peterson and Seligman, 2004). The framework outlines 24-character strengths organized under 6 core virtues, which are considered universally valued across cultures (Peterson and Seligman, 2004). The CSV framework is used to encourage personal growth, well-being, and fulfillment by cultivating these character strengths (Peterson and Seligman, 2004). It is based on the idea that each person possesses these strengths to varying degrees and can develop them to lead a more meaningful and satisfying life (Peterson and Seligman, 2004). One important strength highlighted in the Character Strengths and Virtues (CSV) framework is temperance, which protects against excess and includes traits like humility and modesty. Humility involve allowing one’s accomplishments to speak for themselves, not seeking the spotlight, and not considering oneself more special than one truly is (Peterson and Seligman, 2004). This sense of self-acceptance can lead to happiness and positive behaviors, fostering positive emotions and the confidence to pursue goals (Peterson and Seligman, 2004). Additionally, according to the CSV framework, humility does not necessitate negative self-views or harshly punishing oneself for failures (Peterson and Seligman, 2004). Instead, it embodies non-defensiveness and a willingness to see oneself accurately (Peterson and Seligman, 2004). Peterson and Seligman (2004) note that humble individuals have fewer needs to impress or dominate others. Another important model for understanding the role of humility is the HEXACO model (Ashton and Lee, 2020), which is widely used in personality research. This model is effective in predicting various behaviors, including those related to social interactions, job performance, and moral conduct. A key feature that sets this model apart from others is the Honesty-Humility dimension, which emphasizes traits connected to ethical behavior and personal integrity. According to this model, a humble person would be less manipulative of others, more sincere, fair, modest, as well as less greedy (Ashton and Lee, 2020). These characteristics likely enhance interpersonal relationships, as they make individuals less focused on acquiring power, wealth, or status (Ashton and Lee, 2008). As a result, this shift in focus can lead to greater life satisfaction and improved psychological well-being. Taken together, these personality models, along with the definitions of humility presented in the introduction and the relevance of the second objective of this study, suggest that humble individuals are generally more resourceful, as they can focus on broader and more meaningful values that extend beyond themselves. This focus may enhance the positive impact of HRM practices aimed at promoting psychological well-being, as well as job performance.

Furthermore, the conservation-of-resources model (Hobfoll, 1989) supposes that resource loss or gain results in stress or eustress (i.e., psychological well-being), respectively. Personal characteristics, such as humility, is considered as such a resource. Based on Hobfoll et al. (2018), an abundance of resources creates a “reservoir” that can be filled with individual resources (e.g., humility), as well as organizational resources (e.g., HRM practices). Because individuals strive to obtain, retain, foster, and protect resources (Hobfoll et al., 2018), it could be reasonably anticipated that humble individuals will capitalize on the organizational resources, such as HRM practices, that are made available to them. Note that this is also coherent with the Job-Demands/Resources model (Demerouti et al., 2001) because organizational resources (e.g., HRM practices) expand an individual’s mental capacities, leading to higher psychological well-being and job performance. Inversely, a lack of resources could impair these capacities. Beyond organizational resources, humility, an individual resource, could also play an important role in expanding individuals’ mental capacities, leading to higher psychological well-being and job performance in combination with HRM practices. All of this fits within the person × situation approach of the Job-Demands/Resources model (Bakker et al., 2023). Humility could be useful in both reducing the strain resulting from a lack of HRM practices and boosting the benefits derived from the adequate presence of HRM practices, serving as a vehicle to unleash the effects of HRM practices. Building on this argument, we suggest that humility, when viewed as an individual resource, can be valuable in interaction with HRM practices. This interaction should contribute to employee outcomes, such as psychological well-being and job performance.

In terms of empirical findings, in organizational research, humility has often been studied from the perspective of humble leadership and its effects on employees (See Kelemen et al., 2023, for a review). However, very few studies have examined the effect of humility on oneself as a leader (Yang et al., 2019), and even fewer have examined its effect on oneself as an employee. In this regard, one study found that employees’ humility was associated with their objective job performance via social resources derived from team leaders and colleagues (Li et al., 2021). Moreover, it was demonstrated that students’ humility compensated for a low level of intelligence (i.e., low general mental ability) in terms of individual performance because humility enhances one’s ability to work well with others (Owens et al., 2013) and could also temper the effects of leaders’ narcissism on followers (Owens et al., 2015). Furthermore, it has been established that humble leaders enhance employee well-being via employee humility (Zhong et al., 2020). Similarly, results achieved with a general sample indicate that humility may serve as a predictor of intrinsic aspirations and subjective well-being (Zawadzka and Zalewska, 2019). Humble individuals tend to feel more motivated regarding work and achievement (Rowatt et al., 2006), as well as being more productive at work (Chirumbolo, 2015; Dinger et al., 2015; Rowatt et al., 2006). As for the moderating role of humility, data from a nationwide survey suggest that the magnitude of the negative relationship between stressful life events and measures of well-being (i.e., depressive symptoms, anxiety, happiness, and life satisfaction) was reduced among humble individuals (Krause et al., 2016). Similarly, Chirumbolo (2015) demonstrated that humility functioned as a psychological moderator of the job insecurity effect on counterproductive behaviors at work.

Therefore, we know very little about the effects of employees’ humility on themselves. Humility has been mostly analyzed in terms of leadership abilities. What is not yet well understood is the effect of humility as an individual strength or resource that enables individuals to adapt, cope, or thrive in the workplace. Considering the theoretical and empirical background presented, we propose the following two hypotheses:

*H2*: Humility at T1 plays a moderating role in the relationships between HRM practices at T1 and psychological well-being at T1.

*H3*: Humility at T1 plays a moderating role in the relationships between HRM practices at T1 and job performance at T2.

**Figure 1:**
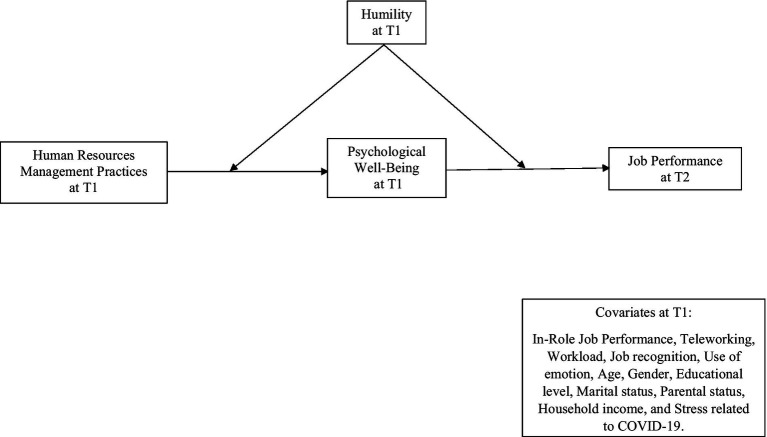
Conceptual model and hypothesis.

See Figure 1, which displays the global hypothetical model.

## 3 Methods

### 3.1 Participants and procedure

This study used data collected via the Leger Opinion (LEO) online panel. Both data collections took place between April 20, 2022, and May 2, 2022, for Time 1 (T1), and between June 20, 2022, and July 3, 2022, for Time 2 (T2). The final sample included 569 workers who filled out the entire questionnaire at both T1 and T2. Based on ethical standards, the participants were asked to review and sign an online informed consent form, and they were briefed about confidentiality before taking part in the research. Aside from a respondent receiving points when completing the survey (note that points can subsequently be exchanged for rewards on Leger Survey’s firm web platform), the participants were not compensated financially. All the workers in this research were eligible to participate (i.e., they were aged 18 years). Because an online panel provided by [Blinded for review] was used, no response rate was available. Our final sample was 51.7% female and had a mean age of 40.3 years.

### 3.2 Measures

#### 3.2.1 Job performance

We followed Williams and Anderson (1991) in measuring in-role performance with a scale that comprised four items (e.g., “I adequately complete the tasks assigned to me”; *α* = 0.94). Each item was scored on a 7-point Likert scale (“Do not agree at all”/ “Very strongly agree”). In-role performance was treated as a continuous variable, with a higher score indicating a higher level of job performance.

#### 3.2.2 Psychological well-being

The World Health Organization (WHO) Well-Being Index (WHO-5) was employed to measure psychological well-being (Heun et al., 2001; Topp et al., 2015), using a scale that comprised five items (e.g., “I have felt cheerful and in good spirits”; α = 0.91). Each item was scored on a 6-point Likert scale (“At no time”/“All the time”). Psychological well-being was treated as a continuous variable, with a higher score indicating a higher level of psychological well-being.

#### 3.2.3 Humility

The Expressed Humility Scale (Owens et al., 2013) was used to measure humility with a scale that comprised nine items (e.g., When I do not know how to do something, I admit it,” “When others have more knowledge and skills than me, I am able to recognize it,” “I am a person willing to learn from others”; *α* = 0.92). Please note that we proceeded to translate these items in accordance with the method proposed by Vallerand (1989). Each item was scored on a 7-point Likert scale (“Do not agree at all”/ “Very strongly agree”). Humility was treated as a continuous variable, with a higher score indicating a higher level of humility.

#### 3.2.4 Human resources management practices

The High Wellbeing and Performance Work System Scale (Parent-Lamarche et al., 2023) was used to measure ten human resources management practices with a scale that comprised 36 items. Each of the following items was scored on a 7-point Likert scale (“Do not agree at all”/ “Very strongly agree”): dotation (e.g., “Are the recruitment and selection processes in this organization impartial (fair and equitable)?”; *α* = 0.82), formation (e.g., “Are extensive training programs provided for me?”; *α* = 0.90), career management (e.g., “Do I have a clear career path (planned promotions) within the organization?”; *α* = 0.79), autonomy (e.g., “Do I have several opportunities to decide how to do my work?”; *α* = 0.89), occupational health and safety (e.g., “Is my work environment safe?”; *α* = 0.90), diversity management (e.g., “Do I feel that management is supportive of cultural differences in this organization?”; *α* = 0.89), performance compensation (e.g., “Does a part of my compensation/salary depend on my individual work performance?”; *α* = 0.83), indirect compensation (e.g., “Does my organization offer me benefits that meet my expectations and needs?”), flexibility (e.g., “Do I have the ability to reduce working hours (e.g., switching from full-time to part-time employment)?”; *α* = 0.80), and performance management (e.g., “Do I receive formal performance feedback from more than one source (i.e., feedback from several individuals such as supervisors, peers, etc.)?”; *α* = 0.95). Human resources management practices were treated as continuous variables, with a higher score indicating a higher level of those practices.

#### 3.2.5 Control variables

Based on the findings of previous research, we controlled for several variables. By controlling for these variables, we were able to better capture the effects of our main variables on psychological well-being and/or job performance. Based on the results of previous studies, we included the following variables: teleworking (Kaltiainen and Hakanen, 2023; Parent-Lamarche, 2022), workload (Jamal et al., 2021; Parent-Lamarche and Boulet, 2021), recognition (Simard and Parent-Lamarche, 2022), the use of emotion (Parent-Lamarche, 2022), age and gender (Dai et al., 2008), educational level, marital status, parental status, household income (Xie et al., 2011), and stress related to COVID-19 (Parent-Lamarche and Boulet, 2021).

A single item was used to measure teleworking (i.e., “I have the opportunity to work at or from home during normal working hours?”), which was coded as a continuous variable, with a higher score indicating more teleworking. This single item was scored on a 7-point Likert scale (“Do not agree at all”/“Very strongly agree”). The effort–reward imbalance questionnaire was used to measure workload and recognition (Siegrist, 1996). Responses were evaluated on a 4-point Likert scale (“Strongly disagree”/“Strongly agree”). Workload consisted of five items (e.g., “I have many interruptions and disturbances while performing my job.”; *α* = 0.81). Recognition was evaluated based on five items (e.g., “I receive the respect I deserve from my colleagues”; *α* = 0.82). Use of emotion was measured based on the Wong and Law Emotional Intelligence Scale, which comprised four items (e.g., “I always set goals for myself and then try my best to achieve them”; *α* = 0.87), and it was coded as a continuous variable, with a higher score indicating the increased use of emotion. Each item was scored on a 7-point additive scale (“Very strongly agree”/“Do not at all agree”). Age was calculated based on the number of years a person had lived. Gender was coded as either 0 (“Male”) or 1 (“Female”). Marital status was coded as 0 (“Single”) or 1 (“Living as part of a couple”). Parental status was evaluated based on the number of minor children living with the participant at the time of the data collection. More precisely, a situation in which no children were living with the participant was coded as 0, and a situation in which any number of minor children (aged below 18 years) were living with the participant was coded as 1. Educational level was based on the highest academic level obtained and comprised ten categories that referred to the number of years necessary to obtain each level, from the lowest number to the highest (1 = none, 2 = high school, 3 = professional school, 4 = college (general), 5 = college (technical), 6 = university (undergraduate certificate), 7 = university (bachelor’s degree), 8 = university (graduate diploma), 9 = university (master’s degree), and 10 = university (doctorate)). Household income was computed before tax deduction and based on the income earned in the year preceding the research (1 = less than $20,000, 8 = $140,000 or more). Stress related to the COVID-19 pandemic was measured using with a single item: “How has the COVID-19 crisis affected your stress level?” Participant responses were coded as either 0 (“The COVID-19 crisis decreased my stress level or did not change my stress level”) or 1 (“The COVID-19 crisis increased my stress level”).

### 3.3 Data analysis

Multiple regression, mediation, and moderation analyses with a robust maximum likelihood estimator to estimate all models were conducted with the 8^th^ version of MPlus software (Muthén and Muthén, 2017), following Preacher and Hayes (2004) method. The goodness of fit was established with the Tucker–Lewis index and the comparative fit index. Values greater than 0.90 and 0.95 are considered indicative of satisfactory and excellent fits, respectively (Hoyle, 1995). This study includes two-time measures administered at a two-month interval. The time lag was chosen according to Meier and Spector (2013) recommendation. First, our analytical procedure was to evaluate a model that comprised human resources management practices at T1 and humility at T1 so that we could test their main effects on job performance at T2 and psychological well-being at T1 (note that this first step was not associated with the empirical validation of the hypotheses). Second, HRM practices at T1 were entered into a second model to examine whether they indirectly influenced job performance at T2 via psychological well-being at T1. Third, we estimated whether humility at T1 had a moderating effect on the relationships between HRM practices and psychological well-being at T1, as well as the longitudinal relationships between HRM practices and job performance at T2. To do so, we introduced, one by one, interactions between humility and HRM practices at T1. In total, 20 moderation effects were tested—one for each HRM practice on psychological well-being at T1, as well as one for each HRM practice on job performance at T2. Given the number of moderation effects to be separately tested, we applied a Bonferroni correction to the estimated moderation effects and set the significance level at *p* < 0.003. Also note that all tested models included the control variables at T1, as well as job performance at T1.

## 4 Results

### 4.1 Descriptive and correlational analysis

Table 1 displays the descriptive results for the research variables (mean/proportion, standard deviation), as well as the correlational results.

**Table 1:** Descriptive statistics and correlations.

		M/%	SD	1	2	3	4	5	6	7	8	9	10	11	12	13	14	15
1	In−role job performance at T2	23.95	4.30	1														
2	In−role job performance at T1	23.87	4.50	0.60**	1													
3	Psychological well−being at T1	16.31	5.09	0.23**	0.25**	1												
4	Humility at T1	48.69	9.28	0.48**	0.67**	0.31**	1											
5	Dotation at T1	12.83	4.34	0.22**	0.23**	0.30**	0.32**	1										
6	Formation at T1	14.93	6.66	0.10*	0.11**	−0.25**	0.27**	0.42**	1									
7	Carrer management at T1	11.13	4.51	0.12**	0.13**	0.32**	0.27**	0.46**	0.66**	1								
8	Autonomy at T1	14.42	4.23	0.27**	0.40**	0.27**	0.41**	0.42**	0.33**	0.45**	1							
9	Occupational health and safety at T1	20.57	5.64	0.27**	0.42**	−0.47**	0.41**	0.50**	0.33**	0.44**	0.56**	1						
10	Diversity management at T1	15.50	4.27	0.30**	0.42**	0.34**	0.50**	0.52**	0.39**	0.43**	0.55**	0.62**	1					
11	Performance compensation at T1	22.09	10.36	0.15**	0.13**	−0.05**	0.02	−0.20**	−0.27**	−0.32 **	−0.16**	−0.16**	−0.09	1				
12	Indirect compensation at T1	14.82	5.17	0.25**	0.32**	0.27**	0.37**	0.28**	0.37**	0.35**	0.29**	0.37**	0.41**	0.01	1			
13	Flexibility at T1	12.67	6.41	−0.04	−0.07	0.21**	0.10*	0.24**	0.34**	0.38**	0.28**	0.27**	0.23**	−0.34**	0.15**	1		
14	Performance management at T1	22.09	10.36	0.07	0.07	0.25**	0.27**	0.38**	0.60**	0.62**	0.34**	0.41**	0.41**	−0.39**	0.43**	0.49**	1	
15	Teleworking at T1	3.78	2.41	−0.01	0.03	0.06	0.13**	0.23**	0.27**	0.27**	0.19**	0.34**	0.21**	−0.20**	0.30**	0.39**	0.45**	1
16	Workload at T1	13.67	3.50	−0.02	0.04	−0.11**	0.09*	−0.04	0.10*	0.02	0.02	−0.15**	−0.04	−0.05	−0.02	0.05	0.03	0.05
17	Job recognition at T1	15.18	3.29	0.25**	0.31**	0.40**	0.40**	0.41**	0.34**	0.45**	0.49**	0.55**	0.55**	−0.12**	0.30**	0.23**	0.44**	0.18**
18	Use of emotion at T1	20.89	4.63	0.41**	0.61**	0.41**	0.65**	0.24**	0.21**	0.30**	0.37**	0.33**	0.36**	−0.04	0.28**	0.05	0.24**	0.05
19	Age at T1	40.31	11.64	0.16**	0.17**	0.11**	0.14**	0.03	−0.10*	−0.06	0.03	0.00	0.03	0.04	0.03	−0.14**	−0.14**	−0.06
20	Gender at T1	0.52	–	0.08*	0.03	−0.03	0.08	0.04	0.06	0.03	0.01	0.05	−0.00	0.07	−0.03	0.00	−0.02	−0.00
21	Educational level at T1	5.30	2.25	−0.05	−0.07	−0.06	0.02	0.01	0.09*	0.03	0.04	0.04	−0.01	−0.04	0.08*	0.00	0.06	0.18**
22	Marital status at T1	0.69	0.46	−0.01	0.00	−0.01	0.03	0.01	0.02	0.02	0.09*	−0.01	0.03	0.03	0.02	0.04	0.01	0.04
23	Parental status at T1	0.40	0.49	0.01	−0.03	0.15**	0.03	0.05	0.05	0.04	0.05	0.01	0.08	−0.04	0.04	0.04	0.05	0.00
24	Household income at T1	5.24	91.92	0.09*	0.14**	0.07	0.18**	0.09*	0.13**	0.12**	0.20**	0.10*	0.15**	0.01	0.25**	−0.00	0.15**	0.20**
25	Stress related to COVID−19 at T1	0.51	–	−0.04	−0.06	−0.22**	−0.01	−0.08	−0.01	−0.06	−0.09*	−0.11**	−0.12**	−0.03	0.02	−0.07	−0.04	0.04

		16	17	18	19	20	21	22	23	24	25
16	Workload at T1	1									
17	Job recognition at T1	−0.08*	1								
18	Use of emotion at T1	−0.08*	0.33**	1							
19	Age at T1	−0.04	−0.00	0.21**	1						
20	Gender at T1	0.02	0.05	−0.02	−0.23**	1					
21	Educational level at T1	0.15**	−0.03	−0.06	−0.08	−0.04	1				
22	Marital status at T1	−0.03	0.04	0.01	−0.11**	0.03	0.11**	1			
23	Parental status at T1	−0.03	0.08	0.04	0.01	−0.06	−0.02	−0.29**	1		
24	Household income at T1	0.06	0.13**	0.12**	0.05	−0.11*	0.31**	0.55**	0.16**	1	
25	Stress related to COVID−19 at T1	0.15**	−0.08	−0.04	−0.05	0.08	0.08	0.03	−0.10*	0.05	1

*p ≤ 0.05; **p ≤ 0.01.

### 4.2 Multiple regression analysis

Although no hypothesis was formulated regarding the direct effects of HRM practices, Table 2 presents the results of our first analytical procedure. Indirect compensation and flexibility at T1 were both directly associated with higher psychological well-being at T1, while performance compensation predicted higher job performance at T2.

**Table 2:** Direct effects of humility and human resources practices management at T1 on psychological well-being at T1 and in-role job performance at T2.

	Psychological well-being at T1	In-role job performance at T2
Constant	17.621**	10.875**
Psychological well-being at T1		
Psychological well-being		0.036
Humility at T1		
Humility	−0.012	0.044
Human resources management practices at T1		
Dotation	0.088	0.078
Formation	0.028	−0.014
Career management	0.053	0.014
Autonomy	−0.090	0.009
Occupational health and safety	0.083	−0.032
Diversity management	0.003	−0.009
Performance compensation	0.036	0.081**
Indirect compensation	0.100*	0.023
Flexibility	0.133**	−0.002
Performance management	−0.037	0.013
Adjustments		
CFI	1.00	
TLI	1.00	
χ^2^ (df)	541.308 (47)**	

*p ≤ 0.05 and **p ≤ 0.01. At T1, the following variables were controlled for: Teleworking, Workload, Job recognition, Use of emotion, Age, Gender, Educational level, Marital status, Parental status, Household income, and Stress related to COVID-19. (unstandardized coefficients).

### 4.3 Mediation analysis

Table 3 shows that psychological well-being at T1 did not play a mediating role between HRM practices at T1 and job performance at T2. In other words, HRM practices at T1 did not predict job performance at T2 via psychological well-being at T1.

**Table 3:** Indirect effects of humility at T1 and human resources management practices at T1 on in-role job performance at T2.

	Estimate	*p*-value
Humility at T1		
Humility	0.000	0.709
Human resources management practices at T1		
Dotation	0.003	0.368
Formation	0.001	0.545
Career management	0.002	0.495
Autonomy	−0.003	0.378
Occupational health and safety	0.003	0.367
Diversity management	0.000	0.964
Performance compensation	0.001	0.485
Indirect compensation	0.004	0.335
Flexibility	0.005	0.308
Performance management	−0.001	0.405

At T1, the following variables were controlled for: Teleworking, Workload, Job recognition, Use of emotion, Age, Gender, Educational level, Marital status, Parental status, Household income, and Stress related to COVID-19 (unstandardized coefficients).

### 4.4 Moderation analysis

**Figure 2:**
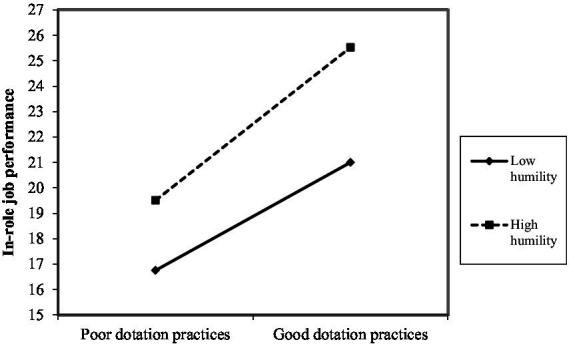
Interaction between humility and dotation practices.

**Figure 3:**
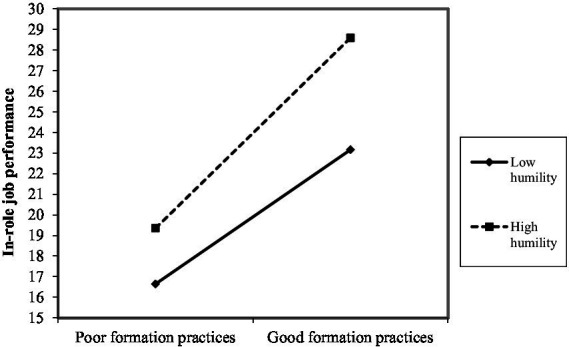
Interaction between humility and formation practices.

**Figure 4:**
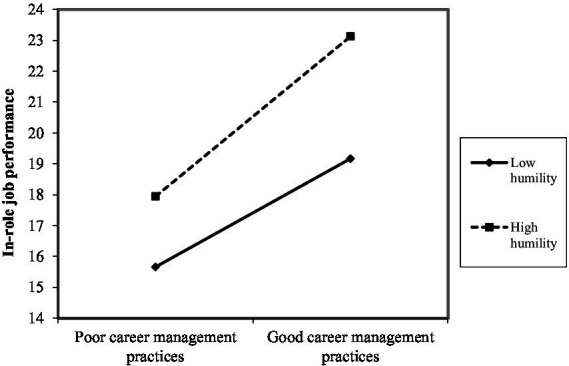
Interaction between humility and career management practices.

**Figure 5:**
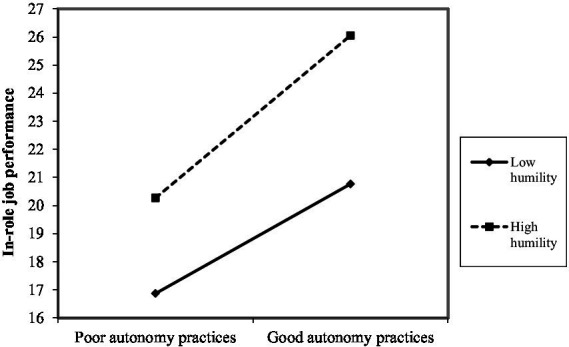
Interaction between humility and autonomy practices.

**Figure 6:**
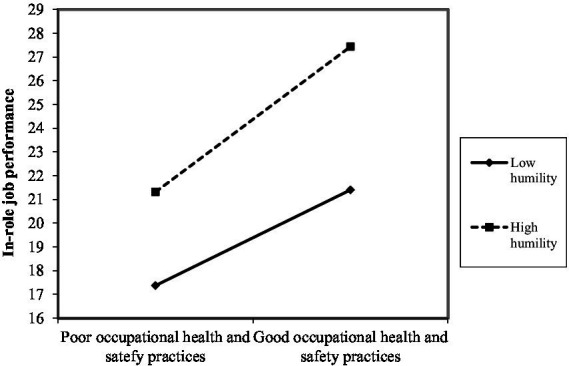
Interaction between humility and occupational health and safety practices.

**Figure 7:**
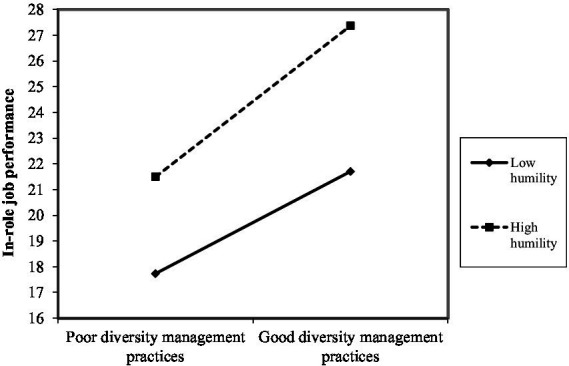
Interaction between humility and diversity management.

**Figure 8:**
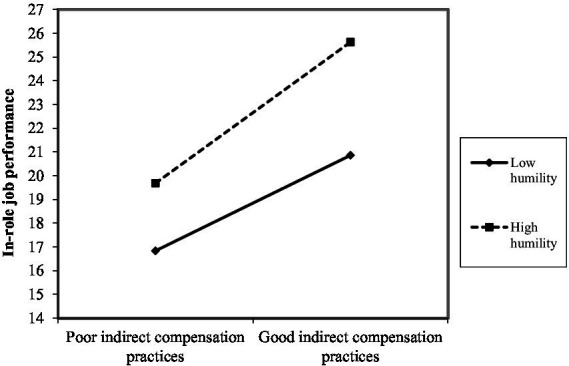
Interaction between humility and indirect compensation practices.

**Figure 9:**
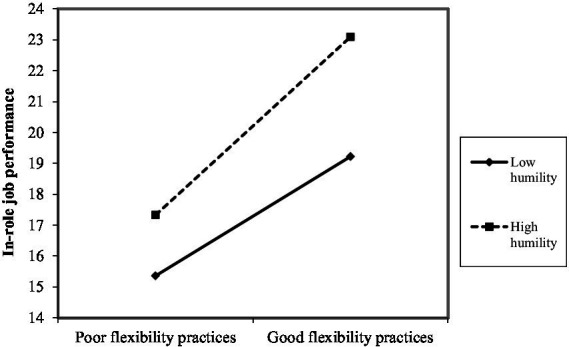
Interaction between humility flexibility practices.

**Figure 10:**
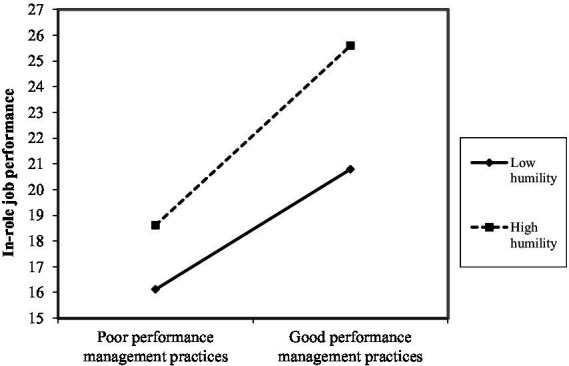
Interaction between humility and performance management practices.

As shown in Figures 2–10, humility did not moderate the relationships between HRM practices at T1 and psychological well-being at T1 but did significantly moderate the longitudinal relationships between HRM practices at T1 (i.e., dotation/Figure 2: (*β* = 0.011, *p* ≤ 0.001), formation/Figure 3: (*β* = 0.010, *p* ≤ 0.001), career management/Figure 4: (*β* = −0.010, *p* ≤ 0.001), autonomy/Figure 5: (*β* = 0.012, *p* ≤ 0.001), occupational health and safety/Figure 6: (*β* = 0.010, *p* ≤ 0.001), diversity management/Figure 7: (*β* = 0.012, *p* ≤ 0.001), indirect compensation/Figure 8: (*β* = 0.014, *p* ≤ 0.001), flexibility/Figure 9: (*β* = 0.008, *p* ≤ 0.001), performance management/Figure 10: (*β* = 0.006, *p* ≤ 0.001)), and job performance at T2. For all significant interactions, the results indicated that when humility was high, the longitudinal effect of good HRM practices led to high in-role job performance. On the other hand, when humility was low, the longitudinal effect of good HRM practices on in-role job performance was low. When both HRM practices and humility were high, in-role job performance was the highest, but when both HRM practices and humility were low, in-role job performance was the lowest. The only HRM practice that was not moderated by humility was performance compensation.

## 5 Discussion

This study was based on a sample of 569 workers in the province of Québec, Canada, and had two objectives. The first objective was to examine the mediating role of psychological well-being in the relationships between HRM practices and job performance. The second objective was to examine the moderating role of humility in the relationships between HRM practices and psychological well-being, as well as between HRM practices and job performance.

The first hypothesis (H1), which postulates that psychological well-being at T1 plays a mediating role in the relationships between HRM practices at T1 and job performance at T2, was rejected. We found that psychological well-being at T1 did not play a mediating role in the relationships between HRM at T1 and job performance at T2. This result is surprising because the prior theoretical and empirical backgrounds point in a different direction. Indeed, the HRM practices that emerged from the integrated mutual-gains model (Guest, 2017; Parent-Lamarche et al., 2023) were supposed to lead to job performance through the psychological well-being of employees, which is coherent with a mutual-gains perspective in general (Peccei and Van De Voorde, 2019). These results force us to consider alternatives to the mutual-gains perspective (win–win scenario), such as the conflicting-outcomes (win–lose scenario) and mutual-losses (lose–lose scenario) perspectives. Skepticism about the idea that HRM practices could move in a direction other than organizational performance at the expense of employee health has already arisen [e.g., Ogbonnaya et al., 2017]. This study’s results may indicate that HRM practices, even those that aim to promote psychological well-being upstream of performance, sometimes fail to do so. Even though HRM practices that aimed to prioritize psychological well-being over performance appear to be promising and optimal, the reality may be more complex. Additionally, the lack of a significant mediating role for psychological well-being may stem from the fact that well-being was measured concurrently with HRM practices. A longitudinal design with three measurement points, as we suggested for future research directions, could potentially yield different results. Indeed, the effects of HRM practices on psychological well-being may not be immediate but may manifest over time. Although no hypothesis was formulated regarding the direct effects of HRM practices, we found that indirect compensation and flexibility at T1 were both directly associated with higher psychological well-being at T1. Therefore, indirect compensation and flexibility appear to be favorable HRM practices that are associated with higher psychological well-being. However, their effects were not strong enough to later influence job performance. Limitations that may partially explained these unexpected results, as well as practical implications will be discussed below.

The second hypothesis (H2), which postulates that humility at T1 plays a moderating role in the relationships between HRM practices at T1 and psychological well-being at T1, was rejected. We found that humility at T1 did not play a moderating role in the relationships between HRM practices at T1 and psychological well-being at T1. This was unexpected because humility is considered a virtue and human strength (Peterson and Seligman, 2004). Individuals who can preserve realistic self-views tend to be psychologically healthy and have high general well-being (Vaillant, 1992). Humble individuals have high self-esteem/ego-strength, which is believed to diminish the experience of significant ego threat and allow them to cope with the limitations of the self (Sandage et al., 2017). This was expected to be associated with high psychological well-being. Additionally, based on the personality models (i.e., Character Strengths and Virtues framework and HEXACO model) presented in the hypothesis development section, along with the definitions of humility outlined in the introduction, it was suggested that humble individuals would be more resourceful, focusing on broader and more meaningful values beyond themselves, thereby amplifying the positive effects of HRM practices designed to enhance psychological well-being. It was also expected that their sense of self-acceptance would lead to happiness and positive behaviors, fostering positive emotions (Peterson and Seligman, 2004). Furthermore, in coherence with the conservation-of-resources model (Hobfoll, 1989), individual resources (e.g., humility) should result in eustress (i.e., well-being). Additionally, an abundance of resources creates a “reservoir” that can be filled with individual resources (e.g., humility), as well as organizational resources [e.g., HRM practices (Hobfoll et al., 2018)], to foster psychological well-being. This was also in alignment with the Job-Demands/Resources model (Demerouti et al., 2001) because organizational resources (e.g., HRM practices) and individual resources (e.g., humility) should expand an individual’s mental capacities, leading to higher psychological well-being. Furthermore, it was empirically established that humble leaders enhance employee well-being via employee humility (Zhong et al., 2020). Similarly, results achieved with a general sample indicate that humility may serve as a predictor of intrinsic aspirations and subjective well-being (Zawadzka and Zalewska, 2019). Also, the magnitude of the negative relationship between stressful life events and measures of well-being was reduced among humble individuals (Krause et al., 2016). Our surprising results suggest that humility could have a darker side, as suggested by Weidman et al. (2018). These researchers suggest that humility can take an appreciative (i.e., elicited by personal success) or self-abasing (i.e., elicited by personal failure) form. Additionally, a previous study also concluded that humility was not related to well-being (Aghababaei and Arji, 2014). These authors explain this unexpected result by noting that, while humility is essential for understanding various social behaviors and interpersonal outcomes, it may not be a key factor in personal pleasure and comfort as typically defined by happiness or psychological well-being. Moreover, another study found that psychological well-being predicted an increase in humility over time, but humility did not predict changes in psychological well-being (Tong et al., 2019). The results indicate that humility does not automatically result in more enjoyable or fulfilling experiences; instead, psychological well-being plays a role in cultivating humility (Tong et al., 2019). Humble individuals are often more attuned to their personal weaknesses, which could potentially undermine their well-being. This serves as an alternative explanation identified in a study by Snow (1995). Another explanation consistent with this last point is that humility often entails a lowering of one’s self-evaluation rather than an elevation (Peterson and Seligman, 2004). This is because humility requires a willingness to see oneself accurately rather than striving for absolute accuracy (Peterson and Seligman, 2004). However, the empirical literature showed us that very little is known about the effects of employees’ humility, as an individual strength or resource that allows to better adapt/cope at work to enhance well-being, on themselves. Future research is needed, and this will be discussed further below.

The third hypothesis (H3), which postulates that humility at T1 plays a moderating role in the relationships between HRM practices at T1 and job performance at T2, was partially supported. Humility played a moderating role in the longitudinal relationships between nine HRM practice at T1, namely dotation, formation, career management, autonomy, occupational health and safety, diversity management, indirect compensation, flexibility, and performance management and job performance at T2. Only performance compensation was not moderated by humility. Note that even though no hypothesis was formulated in this regard, we found that performance compensation at T1 predicted higher job performance at T2. The results obtained were as expected because humility is seen as a predictor of human excellence and flourishing (Peterson and Seligman, 2004). Additionally, humble individuals possess the confidence needed to pursue their goals effectively, in part because humility does not require negative self-views or harsh self-criticism in the face of failures (Peterson and Seligman, 2004). In the same line of thought, the HEXACO model suggests that humility is associated with higher job performance (Ashton and Lee, 2020). Humble individuals attempt to gain authentic or accurate reflections of themselves via others by being transparent about their strengths and limitations (Owens et al., 2013). They also have balanced perceptions that recognize both strengths and limitations, and they do not under-or over-represent themselves (Morris et al., 2005). They also intend to approach interpersonal interactions with the objective of learning via others, which is manifested in showing openness to retroaction, advice, and alternative ideas (Owens et al., 2013), which can help improve performance. Specifically, expressed humility reflects receptiveness to retroaction, better-informed decisions about the attributes needed to meet task performance expectations, and an appreciation of others’ strengths. As anticipated and in accordance with Hobfoll et al. (2018) premise that individuals strive to obtain, retain, foster, and protect resources, it seems that humble individual benefit more strongly from organizational resources such as good HRM practices. Indeed, for all significant interactions, the results indicated that when humility was high, the longitudinal effects of good HRM practices lead to high in-role job performance. Similarly, the Job-Demands/Resources Model (Demerouti et al., 2001) postulates that organizational resources (e.g., HRM practices) and individual resources (e.g., humility) expand an individual’s mental capacities, leading to higher psychological performance. Our results seem to indicate that humility can be useful in both reducing the strain resulting from a lack of HRM practices and boosting the benefits derived from the adequate presence of HRM practices, serving as a vehicle to unleash the effects of HRM practices on job performance. The empirical findings are also in accordance with prior research because one prior study found that students’ humility compensated for a lower level of intelligence (i.e., lower general mental ability) in terms of individual performance (Owens et al., 2013). Humble individuals have the tendency to feel more motivated for work and achievement (Rowatt et al., 2006), as well as being more productive at work (Chirumbolo, 2015; Dinger et al., 2015; Rowatt et al., 2006). Similarly, Chirumbolo (2015) demonstrated that humility functioned as a psychological moderator of the job insecurity effect on counterproductive behaviors at work.

In total, in the absence of a significant moderating effect on psychological well-being, we believe that the component linked to the desire to learn (i.e., teachability) among humble individuals is important in explaining the significant results linked to job performance instead of psychological well-being. Teachable individuals tend to seek out learning opportunities, which likely leads to improved performance. However, this trait may not have a direct impact on psychological well-being. According to Peterson and Seligman (2004), humility plays a key role in teachability, as humble individuals are more open to receiving accurate feedback about themselves. This openness enhances their ability to learn, supporting the idea that teachability is a significant factor in the performance outcomes observed in this study. Therefore, the interaction between HRM practices and humility leads to improved job performance but not psychological well-being. All the same, these studies are relevant to practice.

### 5.1 Theoretical implications

Our main theoretical contribution lies in integrating various models to enhance the understanding of organizational and individual dynamics. By considering both contextual factors and individual characteristics, we can better grasp how individuals feel and react, allowing for more effective adaptation of organizational practices. Additionally, we have synthesized models from different research fields to create a comprehensive framework that incorporates these diverse aspects. The integration of literature on HRM and humility has illuminated the HRM-performance pathways, suggesting that both HRM practices and humility play significant roles in fostering a high-performing workforce. Additionally, this study offers empirical insights into Guest’s theoretical model, revealing that the anticipated win-win scenario is not fully supported. Consequently, our research extends Guest’s model by demonstrating that HRM alone does not appear to significantly impact psychological well-being or performance. However, when humility is included in the equation, performance improves. This finding emphasizes the importance of recognizing employees’ humility as a valuable resource, rather than solely relying on external factors such as leadership or the presence of humble leaders.

### 5.2 Practical implications

This study highlights the importance of HRM practices and humility for human resource (HR) practitioners (or employers if there is no HR department) to target because they were found to be determinant of psychological well-being (only indirect compensation and flexibility who played a direct role) and, more importantly, job performance. Consequently, HR practitioners should ensure that good HRM practices are put in place. On its own, performance compensation is conducive to high job performance, while indirect compensation and flexibility are associated with high psychological well-being. For their part, dotation, formation, career management, autonomy, occupational health and safety, diversity management, indirect compensation, flexibility, and performance management, in combination with humility, can boost job performance over time. Accordingly, efforts should also be directed toward programs that aim to enhance employee humility. For example, practitioners could consider a humility workbook intervention because it has been shown to be effective in increasing humility over time (Lavelock et al., 2014). Also, writing exercises could be considered because it has been proven that they can increase one’s humility by asking one to recall humbling events and write them down (Wright et al., 2017). In addition, organizations could focus on hiring humble leaders, as well as training them (He et al., 2023), considering the fact that humble leaders can foster humility in teams and subordinates (Rego et al., 2017). Organizations ought to encourage leader humility by establishing a culture that values humility and implementing training for leaders (Zhong et al., 2020). Leaders should be supported in recognizing and admitting their limitations, and role reversal between leaders and followers should be promoted (Zhong et al., 2020). This requires organizations to appreciate the acknowledgment of weaknesses and past errors instead of punishing these actions (Owens et al., 2019). Additionally, as suggested by Cuenca et al. (2022), it is possible to cultivate humility among organizational members, particularly employees, by fostering a humble organizational culture. To achieve this, the organization should tolerate mistakes made by both employees and leaders, support open and honest communication, encourage members to be more transparent about their limitations, prioritize employee development, and recognize and appreciate their contributions. Furthermore, employee assistance programs can also be useful to the extent that sessions with clinical psychologists are offered. Ego issues and humility can be addressed in therapy (Horner, 1995; Masterson, 1993). At the same time, meditation and yoga sessions can be offered in the workplace. Humility being a fundamental value that is cultivated within the practice of these activities (as well as cultivating self-care/appropriate self-love), which allows self-enhancement (Gebauer et al., 2018). This will be even more important in the future because of the accelerated changes that will complexity the world of work that lead to organizations having a greater need for leaders and employees who have the willingness, as well as the ego-strength, to acquire new skills and learn from others without feeling threatened.

### 5.3 Strengths, limitations, and future research directions

This study relied on a longitudinal sample (i.e., two-time measures and a panel of 569 participants). Despite this strength, it has several limitations that should be underlined. First, given that all data were collected from the same source (i.e., workers), the possibility of common method bias needed to be considered, particularly due to the perceptual and self-reported nature of our data (Podsakoff et al., 2003). To assess this, we employed Harman’s single factor test (Fuller et al., 2016), as suggested by Podsakoff et al. (2003), when the source of the common factor is unspecified and cannot be directly measured. We performed a factor analysis in SPSS, loading all variables and examining the unrotated solution to check whether a single factor explained most of the total variance. Our results showed that the highest loading factor accounted for 35.81% of the total variance. Since this is below the 50% threshold, we concluded that common method bias is minimal and does not hinder the validity of our subsequent analyses. Second, humility was assessed by the respondents themselves, which suggests that the responses given could be biased in terms of the perception and understanding of the questions asked, as well as being tainted by a desire to respond in a socially desirable manner. Indeed, relying on self-reported humility presents a significant limitation, as it is susceptible to social desirability bias (e.g., Lee and Ashton, 2012). Consequently, future research should consider triangulating these data with assessments from peers or supervisors (Ashton and Lee, 2020). The same applies to questions about HRM practices, even though we believe that employee perception is paramount (i.e., a good HRM practice will presumably have an effect only if it is perceived as such). Accordingly, future research should combine various measures of humility (e.g., the reports of colleagues and supervisors), as well as HRM practices. Furthermore, future research should investigate the effects of the different dimensions of the Expressed Humility Scale: (1) accurate self-awareness, (2) appreciation of others’ strengths and contributions, and (3) teachability. This is especially relevant given the surprising results obtained in the present study. It is conceivable that the outcomes may vary depending on these distinct sub-dimensions. Moreover, our study did not integrate personality frameworks like HEXACO, which could provide deeper insights into the multifaceted nature of humility. Future research should investigate how the Honesty-Humility trait influences both psychological well-being and performance in various organizational contexts. Indeed, the concept of humility can be viewed either as a personality trait or as a behavior that stems from specific personality profiles, a distinction that future research should explore in greater depth. In this study, we adopted the Expressed Humility Scale, which frames humility as a set of behaviors, rather than a stable personality trait. On the other hand, the Honesty-Humility scale from the HEXACO personality model considers humility to be a more consistent and enduring personality trait, applicable across various situations. Cloninger’s work (e.g., Cloninger et al., 1993) provides yet another perspective by examining traits closely related to humility, such as self-transcendence and cooperativeness. These dimensions, which include qualities like altruism, openness to others, and the ability to perceive oneself as part of a larger whole, offer a more nuanced understanding of humility as a behavioral expression shaped by certain combinations of personality traits. In summary, Cloninger suggests that traits such as cooperativeness and self-transcendence are crucial in shaping how humility is expressed in everyday behaviors. This highlights the need for future research to integrate these different frameworks to fully understand the dynamic nature of humility—whether as a stable trait or a behavior influenced by context and personality composition. Third, our study relied on self-reported data obtained via [Blinded for review]. Consequently, we do not have information regarding the employers’ participants (e.g., organization size, sector, job positions, and the presence of a union). This could have been helpful in better interpreting the results obtained. Thus, future research should attempt to collect data across multiple organizations from different sectors/industries and of different sizes to better contextualize and interpret the identified effects. Fourth, the HRM practices measured in this study were derived from a new scale named the High Wellbeing and Performance Work System (HWBPWS), which was based on the integrated mutual-gains model of Guest (2017) and developed and validated by Parent-Lamarche et al. (2023). The quasi-absence of significant results for psychological well-being may be due to the HRM practices most conducive to psychological well-being being those that are adapted to organizations’ and employees’ particularities. This is consistent with a previously presented point indicating that organizational characteristics should be considered in future studies, in addition to alternative individual characteristics. Indeed, the person x situation approach of the Job-Demands/Resources model should still be considered (Bakker et al., 2023). Furthermore, the quasi-absence of significant results may also be because psychological well-being was measured at T1 (i.e., the same time point as HRM practices). This can be problematic, as we do not know how long these practices have been in place. To overcome this limitation, future research should include three measurements, measuring HRM practices at Time 1, psychological well-being at Time 2, and job performance at Time 3. Then, it will be possible to ensure that HRM practices can be deployed long enough to impact psychological well-being and that this can impact performance subsequently over time. Fifth, taking into consideration the limitations enumerated, the results obtained from this study may not be generalizable. Sixth, gender and age were controlled for in this study, but the effects of HRM practices and humility could be different for men and women, as well as for different age groups. Additionally, alternative moderators and/or mediators should be considered in the sequence we tested. We did not investigate alternative pathways through which HRM practices may impact performance via psychological well-being. Seventh, future research could also explicitly address the capacity for humility to develop and expand in employees. Paying attention to the egos of others and working to cultivate one’s own should be mutually self-reinforcing in terms of creating a healthy work environment filled with humility, thereby promoting job performance. Eight, our study did not consider the complexity of measuring work performance. This complexity is reflected in the heuristic conceptual framework of individual work performance, which encompasses four dimensions: task performance, contextual performance, adaptive performance, and counterproductive performance (Koopmans et al., 2011). Future research should embrace multifaceted approaches to effectively measure and predict work performance.

## 6 Conclusion

In this study, human resources management was expected to have a favorable effect on job performance via employee psychological well-being according to a mutual-gains perspective (win–win scenario). Instead, the results obtained seem to indicate that none of the three previous scenarios (win–win, win–lose, and lose–lose) is validated. In consequence, the relationships between HRM, psychological well-being, and job performance remain poorly understood. The criticism that HRM practices influence job performance directly, without affecting employees’ psychological well-being, remains legitimate. On the other hand, our study makes an important contribution regarding the effect of humility in this regard. Indeed, the effects of HRM practices on job performance are much greater when we consider employee humility. In fact, this study demonstrated that job performance did differ among workers facing the same HRM practices. Therefore, a person must express the humility “bend” to avoid the collapse of an ego “break” and the anticipated consequences for job performance but not psychological well-being. Still, employers should deploy practices that can allow their employees to grow individually and develop their own resources and strengths, especially in the current competitive and, therefore, potentially ego threatening, work environment. Humility should help expand the window of tolerance to criticism and the acceptance of constructive retroaction, which commonly occurs in work environments.

## Data Availability

The raw data supporting the conclusions of this article will be made available by the authors, without undue reservation.
